# A Neurophysiological Evaluation of Cognitive Load during Augmented Reality Interactions in Various Industrial Maintenance and Assembly Tasks

**DOI:** 10.3390/s23187698

**Published:** 2023-09-06

**Authors:** Faisal M. Alessa, Mohammed H. Alhaag, Ibrahim M. Al-harkan, Mohamed Z. Ramadan, Fahad M. Alqahtani

**Affiliations:** Department of Industrial Engineering, College of Engineering, King Saud University, P.O. Box 800, Riyadh 11421, Saudi Arabia

**Keywords:** augmented reality (AR), maintenance task complexity, cognitive workload, human performance, electroencephalography (EEG)

## Abstract

Augmented reality (AR) has been shown to improve productivity in industry, but its adverse effects (e.g., headaches, eye strain, nausea, and mental workload) on users warrant further investigation. The objective of this study is to investigate the effects of different instruction methods (i.e., HoloLens AR-based and paper-based instructions) and task complexity (low and high-demanding tasks) on cognitive workloads and performance. Twenty-eight healthy males with a mean age of 32.12 (SD 2.45) years were recruited in this study and were randomly divided into two groups. The first group performed the experiment using AR-based instruction, and the second group used paper-based instruction. Performance was measured using total task time (TTT). The cognitive workload was measured using the power of electroencephalograph (EEG) features and the NASA task load index (NASA TLX). The results showed that using AR instructions resulted in a reduction in maintenance times and an increase in mental workload compared to paper instructions, particularly for the more demanding tasks. With AR instruction, 0.45% and 14.94% less time was spent on low- and high-demand tasks, respectively, as compared to paper instructions. According to the EEG features, employing AR to guide employees during highly demanding maintenance tasks increased information processing, which could be linked with an increased germane cognitive load. Increased germane cognitive load means participants can better facilitate long-term knowledge and skill acquisition. These results suggested that AR is superior and recommended for highly demanding maintenance tasks since it speeds up maintenance times and increases the possibility that information is stored in long-term memory and encrypted for recalls.

## 1. Introduction

The overall complexity of maintenance and assembly tasks grows as product mass customization increases [1,2]. The number of items offered by manufacturing enterprises has dramatically increased as a consequence of increased product saturation, competition, worldwide commerce, and growing demand for diverse sorts of products [3,4]. As a result, the complexity of maintenance tasks may rise, potentially leading to decreased operational effectiveness [5]. Task complexity in terms of the required cognitive resources is defined as “the difficulty in human information processing” [6], which is mainly affected by system design or environmental differences. The structure of new equipment and the environment where maintenance specialists accomplish their responsibilities are often very complex. Maintenance specialists are crucial individuals within the system of the manufacturing industry, in which they study the status of the maintenance goal, identify faults, and undertake maintenance tasks. Such responsibilities often involve a large amount of information processing, which contributes to elevated levels of cognitive workload. Hence, a maintenance activity is more complicated if it involves making decisions that are accompanied by high cognitive demands. Furthermore, new technologies and techniques have been continuously adapted in the manufacturing environment as a response to the continuous efforts to increase productivity [2]. Thus, maintenance specialists are required to adapt and learn new techniques to accomplish their duties, which poses a potential increase in task complexity and, thus, could impose high mental demands [7,8,9]. 

Traditionally, instructions for maintenance tasks are delivered to workers on paper with expert assistance [10,11]. However, time, availability, and cost restrictions may prevent expert assistance. In addition, there is a positive relationship between the efficiency of the paper-based instruction method and the complexity of maintenance tasks [12]. To improve maintenance efficiency, it is necessary to provide the correct information about the appropriate quantity at the appropriate time to the appropriate worker [2]. Therefore, a good maintenance support system that can support or aid the human technicians is necessary to reduce financial costs and prevent errors. Such a system should feature an intuitive, timely, and effective training tool to ensure that required abilities and skills are delivered effectively to workers, especially newly hired workers, or when new technologies are adapted [13]. 

The recent developments in interactive multimedia mediums provide promising solutions for the weaknesses and limitations of the traditional paper-based instruction method. For instance, augmented reality (AR) technology is a powerful tool that overlays virtual information produced by computers into a real environment, which promotes experience and interaction [14], as well as efficiently solving maintenance issues caused by the complexity of modern equipment and, thus, improving quality and maintenance effectiveness [15,16,17]. However, most previous studies assessing the effects of AR-based instructions on cognitive load have applied subjective measurement methods and tested tasks that are often unrepresentative of real industrial maintenance settings.

Cognitive load is defined as “the amount of cognitive effort being put into working memory at any particular time” [18]. It is usually connected to the Cognitive Load Theory (CLT), which examines how mental resources are directed and exploited during training and problem-solving. The need for addressing cognitive load during training is based on CLT and on the idea of the human cognitive model, which consists of sensory input, working memory with a limited capacity, and long-term memory with an infinite storage capacity [18,19]. Overall, CLT distinguished three types of memory loads, namely intrinsic (ICL), extraneous (ECL), and germane cognitive loads (GCL) [18]. ICL is associated with the inherent difficulty of the subject matter and the number of interrelated components in the learning task. ECL is linked to the cognitive effort required for the learner to look for/identify task-relevant information and is the result of additional demands generated by poor instructional design. This mental load is non-task-related and should be minimized by optimizing instructions and minimizing unrelated noise [20]. GCL is associated with the cognitive exertion required for learners to accomplish learning objectives or internalize new knowledge in long-term memory (i.e., the construction of schemas) [18]. Thus, the purpose of instructions or guidance should be to assist the development of schemata in working memory without exceeding its capacity with more information [21,22]. As a result, in addition to past knowledge as a learner’s specific causative element affecting the capacity of working memory, instructions for tasks, through which learners gain new information, must be considered when determining working memory capacity. Cho et al. [23] modified the standard CLT model proposed by [24,25] to include the physical learning environment, learner tasks, and their interaction as contributing elements to cognitive load levels. As a consequence, interactions between the environment and the learner, the learner and the task, and the task and the environment may all alter cognitive load [23].

In comparison with traditional training methods, previous researchers focused mainly on the effectiveness of AR as a training or guiding tool based on task completion time and success rate. Previous research has shown that presenting task instructions via an AR display reduced errors and time to complete tasks while maintaining high levels of technology acceptance from operators [26,27,28,29,30,31,32,33]. However, there is a significant lack of empirical evidence from user studies implementing objective measures to evaluate the effectiveness and adverse effects of AR-based instruction methods (e.g., headaches, eye strain, nausea, and mental workload) when compared to paper instruction. For instance, several previous studies have demonstrated the cognitive load and attention of AR-based instruction using subjective measures such as NASA-TLX [32,34,35,36]. Although very useful, NASA-TLX has the potential to be influenced by participant bias [37,38] and fails to provide accurate, real-time cognitive measurements [39]. On the other hand, objective assessment methods could provide a direct and real-time evaluation of the physiological state of individuals during task performance. The recent advancements in the human factors field provide objective assessment methods to investigate the human brain function concerning perceptual, cognitive, and motor functioning in real or simulated industrial settings. Such methods include electroencephalography (EEG), electrocardiogram (ECG), electrooculography (EOG), and functional magnetic resonance imaging (fMRI) [40,41]. 

The electroencephalogram (EEG) is an emerging, noninvasive, affordable, and portable neuroimaging modality used to record voltage fluctuations occurring as ionic current flows inside the brain’s neurons [42,43]. The electrical potential (often measured in microvolts) in the brain is recorded instantaneously by placing various electrodes at different locations on the scalp (regularly positioned over the frontal, parietal, occipital, and temporal brain lobes) [43,44]. Thus, EEG brain signals are susceptible to vigilance variability and are associated with various cognitive states of an individual while performing a specific task [45,46,47,48,49]. Mainly, EEG has been utilized to identify the different features of cognitive performance such as perception, emotions, memory, monitoring, and control [50]. To obtain meaningful data about an individual’s cognitive load, EEG signals combine brain patterns at different levels of frequency which fall into multiple frequency bands (e.g., Delta (δ: 0.5–3.5 Hz), Theta (θ: 4–7 Hz), Alpha (α: 8–12 Hz), Beta (β: 13–30 Hz), and Gamma (γ: 31–50 Hz) [45]. 

Previous studies have utilized EEG trends in various frequency bands to evaluate the cognitive characteristics of performance in different fields. Sassaroli et al. [51] found that frontal lobe activity in brain regions could be used as a measure of mental workload. An increase in prefrontal cortex activation has been connected to an increase in mental workload while driving [52]. Increased mental workload has been linked to theta and alpha powers [53]. The fluctuation of the theta and alpha bands’ power has been linked with memory and complex cognition performance [54,55]. Jensen & Tesche. [56] indicated that the rise in theta activity in the frontal cortex was connected to the increased load on the memory while working on a memory-intensive task. In addition, the complexity of the task has been found to have positive associations with theta power in the frontal lobe [57] and with alpha power when the left and right hemispheres were considered [58]. Puma et al. [59] evaluated the impact of task complexity on theta (θ) and alpha (α) band power and found that the Ɵ and α power spectral densities were increased for those who performed at a high level in comparison to those who performed at a medium or low level. Furthermore, it was discovered that beta (β) band power decreases during new information acquisition [60]. Diaz-Piedra et al. [61] examined the influence of flight difficulty on the activity of the brain. They discovered that as complexity increased, performance declined, and the full range of EEG activity (0.5–30 Hz) demonstrated a rising pattern. Iqbal et al. [62] discovered that Ɵ power has the possibility to detect a discrepancy in the way processes act and the operator’s mental models. They also found that θ power has a positive relationship with operator effort required across process deviations.

This study aims to advance the body of knowledge relative to evaluating cognitive workload while using AR systems in the training of maintenance and assembly tasks. To accomplish this goal, both subjective and objective measurement methodologies are considered to evaluate cognitive workload during real-world representations of maintenance tasks. Therefore, the main objective of this study is to investigate the effects of different instruction methods (AR-based and paper-based instruction) and task complexity on cognitive workload and performance. The cognitive workload is evaluated by the EEG power spectrum density of alpha, beta, and theta in the frontal, central, occipital, temporal, and parietal brain regions and by perceived workload (NASA-TLX). The current study seeks to answer the following question: how do AR-based instruction methods with various demanding tasks affect human cognitive load (i.e., brain activity and perceived workload) and performance (completion time)? It was hypothesized that performing the experimental maintenance tasks with AR-based instructions would be linked with a higher cognitive load as participants must simultaneously attend to information in both real and virtual form. Yet, it was also anticipated that AR-instructions would improve performance time since they facilitate easier information availability as compared to traditional instructions.

## 2. Materials and Methods

### 2.1. Apparatus

The maintenance tasks were performed on a piston pump subsystem. This piston pump is part of the “GUNT” Practice Line for assembly, maintenance, and repair, which is designed for students and technicians’ training (set: MT 184, Assembly & Maintenance Exercise: Piston Pump). A screwdriver, a wrench, a soft hammer, a jig, and a bearing puller were all needed to complete the tasks on the piston pump. Two instruction methods (Microsoft HoloLens and paper instructions) were used to guide the participants to complete the maintenance tasks. The maintenance instructions for the two tasks were communicated via the Microsoft HoloLens AR system. The paper instruction manuals for the two tasks were developed based on the manual provided by the manufacturer (Gunt technology Ltd., Barsbüttel, Germany). A Live Amp wireless amplifier (Brain Products, GmbH, Gilching, Germany) with 32 channels (weighing 60 g) was used for amplifying and digitizing EEG signals. To hold the EEG electrodes, a black sub-inion cap (EASYCAP, Brain Products GmbH, Gilching, Germany) with an integrated chin made of soft, high-comfort fabric was used. A brain vision recorder system (Brain Products GmbH, Gilching, Germany) was used for recording EEG signals.

### 2.2. Participants

Twenty-eight healthy male university students with a mean age of 32.12 (SD 2.45) years from King Saud University, Riyadh, were recruited via opportunity sampling. The minimum, maximum, and median ages of the participants were 27, 35, and 32.5 years, respectively. A self-reported screening survey was adapted to exclude participants with a pre-existing orthopedic surgery, nervous system disorder, articular pain, a history of musculoskeletal disorder, injury to the arms, legs, or spine, or a heart and/or lung problem, an allergic reaction to any adhesive and gel materials used in this study, or sleep disturbance in the two weeks prior to the experiment. Moreover, each participant was also asked to avoid eating two hours before training or data collection. Participants were also instructed to have a normal amount of sleep (approximately six hours of sleep each night) and refrain from engaging in any excessive physical activity before the experimental sessions. Participants in the experiment were selected so that they had little to no background information about the task to be performed.

The IRB’s approval for this experiment was obtained from the Human Participants Review Sub-committee, the Institutional Review Board, King Khalid University Hospital, the College of Medicine, and King Saud University. The approved written informed consent form (ethical approval code: E-19-4467) was readied and signed by each participant before participating in the experiment.

### 2.3. Experimental Design

A 2 × (2 × 14) mixed design was implemented to represent the experimental design with one between-subject variable and one within-subject variable. The first independent variable, which was treated as a between-subject variable, is the instruction methods at two levels (i.e., AR-based instructions and paper-based instructions). The second independent variable, which was treated as a within-subject variable, is the task complexity with two levels (i.e., low-demanding task and high-demanding task). The dependent variables were the EEG power spectrum density of alpha, beta, and theta in the frontal, temporal, parietal, and occipital brain regions and the perceived workload (NASA-TLX).

### 2.4. Maintenance Tasks

**Figure 1:**
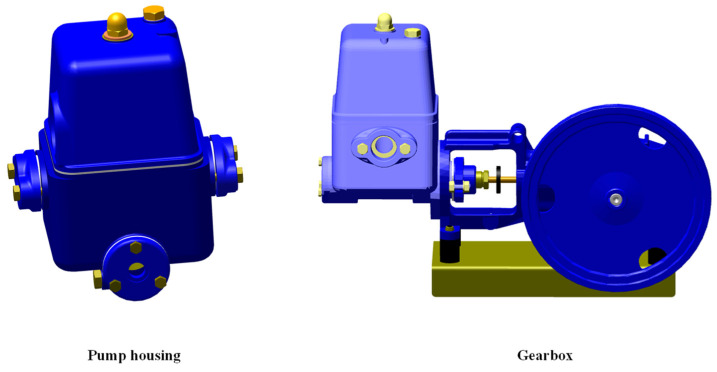
The gearbox and pump housing of the piston pump in a fully assembled state.

The maintenance tasks used in this study are the same as those used in Alhaag et al. [63] study. The overhaul maintenance operations of the piston pump were selected for this study and divided into two maintenance tasks: repairing a piston pump’s gearbox (i.e., a high-demanding task) and checking the seal of the pump housing (i.e., a low-demanding task). Figure 1 presents the gearbox and pump housing of the piston pump in their fully assembled state. In the highly demanding task, participants were required to repair the gearbox of the piston pump and check all seals in the gearbox. The gearbox of the piston pump consists of 32 groups of parts that must be disassembled, maintained, and reassembled over 40 steps using standard tools such as an open-ended wrench, screwdriver, puller, jig, and soft hammer. Table 1 presents the maintenance steps for this highly demanding task. In the low-demanding task, participants were required to check all seals on the pump housing. Compared to the highly demanding task, this task included fewer steps (26 steps, as shown in Table 2), the use of unspecialized hand tools and lightweight parts, and required less precision and effort. The degree of complexity was evaluated experimentally by Alhaag et al. [63].

### 2.5. Instruction Methods

#### 2.5.1. Paper-Based Instructions Method

**Figure 2:**
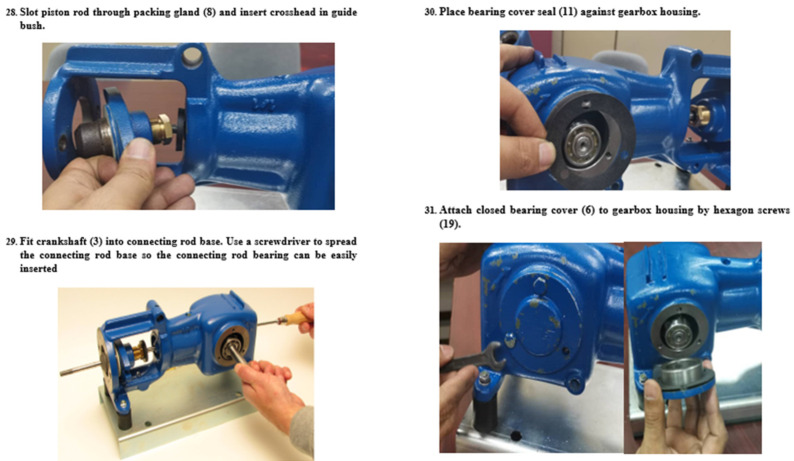
Examples of the paper-based instructions for the highly demanding maintenance task (Gearbox of piston pump).

**Figure 3:**
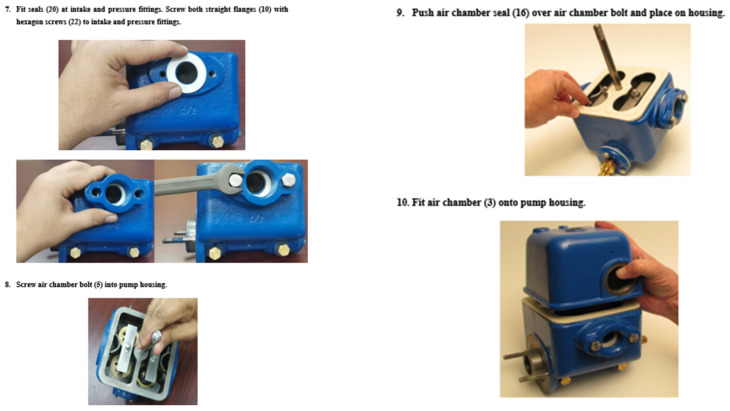
Examples of the paper-based instructions for the low-demanding maintenance task (Pump housing).

The paper-based instructions were extracted from the manual of the piston pump that is used for troubleshooting and repair. For the two maintenance tasks, two paper-based instructions were designed and delivered on A4 sheets of paper, one page per step. Each step was composed of pictures, text information, and the needed tools. For the highly demanding task, the paper-based instructions consisted of 22 pages and 40 steps. The first page contained a list of all parts (disassembled) and their code numbers, as well as the objective of the task. Each of the following pages (page 2 to page 20) contained two of the 40 steps (see Figure 2). The final page contained the full assembly of the gearbox. For the low-demanding task, the paper-based instructions consisted of 14 pages and 26 steps. The first page contained a list of all parts (disassembled) and their code number, as well as the objective of the task. Each of the following pages contained two of the 26 steps (see Figure 3).

#### 2.5.2. AR-Based Instruction Method

The first step in building the AR instructions was to create 1:1 CAD models for the piston pump using the SolidWorks software. After that, PTC Creo illustrate was used to build and edit the sequence animation for the AR experience. The 3D instructions for assembly, repair, and disassembly of the two tasks were created based on visual cues and animations. Step-by-step instructions were created using several motion effects (as applicable) for individual steps, such as fly-in/fly-out, fade-in/fade-out, shake, pulse, unscrew, etc. These effects were added to each step individually, and then all steps of one intended instruction were combined into one fluid motion. Also, other resources, such as animated hand tools like a screwdriver, wrench, arrow, and puller, were added. Then, the 3D instructions were published as a PLZ file to be imported by Vuforia Studio software to create the AR experience. Vuforia Studio is a web-based tool used to create and publish AR experiences and enables users to provide step-by-step work instructions and overlay 3D digital content on real-world equipment to provide contextual knowledge. In addition, the programming language JavaScript was used to develop the key features of the applications.

**Figure 4:**
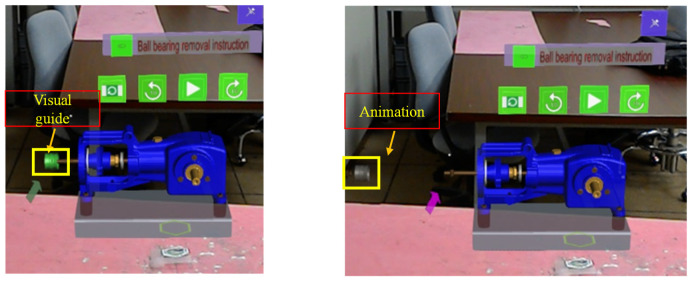
Samples of AR instruction for removing duo-piston during gearbox maintenance as viewed by Microsoft HoloLens.

Then, the created experiences for the maintenance tasks were published to the cloud-based “Vuforia Experience Service” with quick response (QR) codes and Things. Vuforia Experience Service represents a server used to store and deliver published AR experiences, which allow users to access them on Microsoft HoloLens 1 devices through the Vuforia View application. For displaying the AR instruction, the Vuforia View app stored on Microsoft HoloLens was used for scanning a specific Thing marker placed on the maintenance workplace 130 cm away from the participant. After the Thing Mark was identified, Microsoft HoloLens overlaid a 3D CAD model of the selected task on the user’s surroundings. In this method, the maintenance steps were presented as 3D animated models in the AR environment. The participants can interact with the 3D contents and control the playback of the animation by directing their gaze to the virtual buttons (e.g., play, next step, previous step, reset, name of tasks) placed above the 3D model and making a pinching gesture with their fingers. If the participant clicks the play button, a step-by-step animated sequence for the selected task is displayed. Figure 4 presents examples of AR instructions for one step (removing the duo-piston in gearbox maintenance).

### 2.6. Cognitive Load Measurement

#### 2.6.1. Electroencephalography (EEG) Signal Response

**Figure 5:**
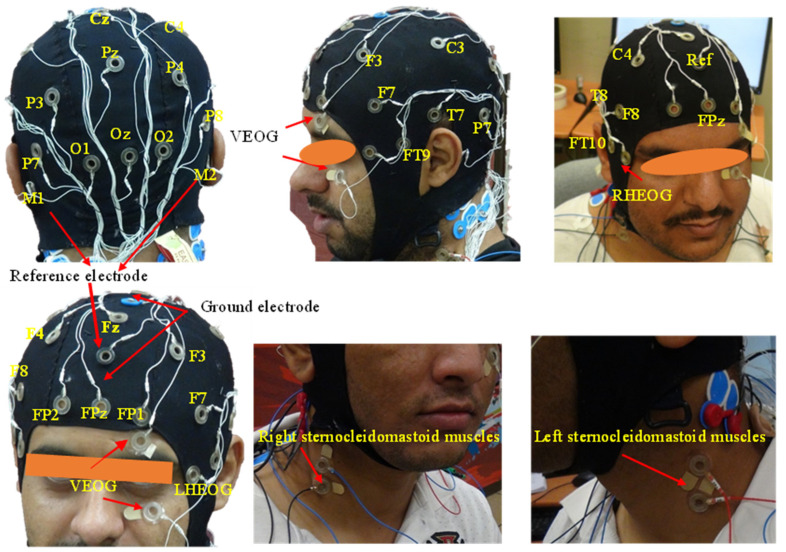
EEG, EOG, and EMG Electrodes placement.

To record the EEG signals, a standard protocol was followed to place the electrodes on the scalp based on the international 10–20 placement system [44]. A Live Amp with 32 channels (24 EEG, 3 EOG, 2 EMG, and 3 head acceleration) and a wireless amplifier (Brain Products GmbH, Germany) were used to record EEG, EOG, and EMG signals. An ActiCAP with 24 mounted Ag/AgCl electrodes placed on the scalp was used to record EEG activities from five brain regions, including the frontal for attentiveness, reasoning, and motor planning; parietal regions for mental processing; temporal for hearing and remembrance; central for sensorimotor function; and occipital region for sensory and visual awareness [42,54,64]. For frontal regions, the EEG signals were recorded from the Fp1, Fp2, F7, F3, F4, F8, and Fz sites, as shown in Figure 5. For the parietal regions, the EEG signals were recorded from the P3, Pz, P4, P7, and P8 sites. For the temporal regions, the EEG signals were recorded from the FT9, FT10, T7, and T8 sites. For the central regions, the EEG signals were recorded from the C3, Cz, and C4 sites. For the occipital region, the EEG signals were recorded from the O1, Oz, and O2 sites. Also, four Ag/AgCl surface electrodes were connected to the ActiCAP for recording the vertical and horizontal eye movements. Two electrodes were placed at the right and left outer canthi of the eyes to record the horizontal eye movements (HEOG). The other two were located about 2 cm above and below one eye to log the eye blinks and the vertical eye movements (VEOG) [65,66]. Moreover, for recording EMG signals, Ag/AgCl solid adhesive pre-gelled electrodes connected to ActiCAP were placed on the right and left of the sternocleidomastoid muscles, as shown in Figure 5. The ground electrode was placed at FPz. The vertical and horizontal eye movements (i.e., EOG data) and sternocleidomastoid muscle activities (i.e., EMG data) were used for EEG artifact identification [67,68]. Linked mastoids (M1 and M2) were used as reference electrodes.

The EEG, EOG, and sternocleidomastoid EMG signals were acquired and amplified at a sampling rate of 1000 Hz with the help of a brain vision recorder system (Brain Products GmbH, Germany). The contact impedance between all electrodes and the skin was kept under 20 kΩ. Electrode impedances were adjusted before raw data collection by filling the electrodes with electrolyte gel. Electrolyte gel was applied by syringe to create a stable electrical connection between each electrode and the scalp. The raw EEG data contains artifacts that have non-neural origins and are usually the result of eye movements, eye blinks, jaw movements, and muscle movements. The recorded EEG signals were initially inspected visually for these suspicious artifacts and then preprocessed using EEGLAB [69]. Afterward, power line noise and other high-frequency noise were eliminated using a low-pass four-pole elliptic filter with a cut-off frequency of 50 Hz. Then an independent component analysis filter (ICA) was applied to identify and eliminate the noise and reconstruct the lost data using a spatial mixing matrix under the assumption of volume conduction [70,71].

The EEG data were processed using wavelet packet analysis and decomposed using a six-octave wavelet, with the dB4 mother wavelet being chosen as the most appropriate function [72,73]. The multilevel discrete wavelet transform (DWT) was utilized so that the EEG data could be decomposed into their respective rhythms (i.e., θ, α, and β). To calculate the power density of EEG rhythms from the five regions with a frequency resolution of 1 Hz and a range from 0.5 to 50 Hz, a digital Fast Furrier Transform (FFT)-based power spectrum analysis (using the Welch approach, Hanning windowing function, and 50% shift) was applied [73,74]. The response variables related to the EEG were the power spectrum density (PSD) of the three bands (θ, α, and β) in the frontal, central sulcus, temporal, occipital, and parietal brain regions. These parameters were considered indicators of cognitive processing workload.

#### 2.6.2. Subjective Response

For subjective workload, the NASA-TLX was utilized to assess participants’ perceived workload following each task on six dimensions (i.e., mental, physical, and temporal demands, performance, effort, and frustration) and the overall weighted workload [37]. Participants rated the dimensions on a scale of 0–20 (where 1 represents a low workload and 20 represents a high workload). Also, participants were given a list of six components and asked to compare each pair (for a total of 15 comparisons) to choose which one would be most useful for analyzing cognitive stress. The relative importance of each factor was calculated based on these comparisons. Next, we multiplied the component’s weighted value by its rating to get its total workload.

### 2.7. Experimental Setup and Procedure

The study was conducted in the Industrial Engineering Department’s maintenance lab. The temperature, humidity, and light intensity at the center of the table in the laboratory were 21.4 °C, 18.2%, and 392.5 lux, respectively. The experimental zone was secure from vibrations or strong odors during the task’s execution. To attract participants, an announcement and invitation were issued and distributed at King Saud University. The participants were invited to participate in a pre-test session as well as two test sessions ranging from 1 to 4 h. Upon participant arrival, the purpose of the experiment was explained in detail, and the screening process was completed. The results of the health survey were reviewed and approved; if acceptable, the participant was asked to sign a consent form and fill out a demographic questionnaire. In addition, the participants were requested to provide background information and answer questions related to their existing AR experience. After that, their anthropometric data was collected.

Next, participants were randomly assigned into two groups and given a briefing on how to use the instruction method assigned to their group. The first group received AR-based instructions, in which a Microsoft HoloLens1 was used to display relevant maintenance instructions and guide the participant to perform the maintenance tasks. In the AR-based instruction group, participants were given 10 to 15 min to familiarize themselves and learn how to handle, adjust, the HoloLens and interact with it [9,75]. Participants were also familiarized with using the toolbox, such as a screwdriver, bearing puller, jig, etc. The second group received paper-based instructions, in which A4 paper documentation was used to deliver the maintenance instructions and guide the participants to perform the maintenance tasks. The paper-based group was familiarized with using the toolbox and reading the document. Once the participants felt confident using the Microsoft HoloLens or the paper instructions, they were asked to leave the lab and return the next day to start the actual training session.

The participant returned to the lab the next day to perform the maintenance tasks with the help of the assigned instruction method from the previous day (AR-based or paper-based instructions). On this day, for example, participants in group 1 (the AR-based instruction group) were randomly assigned to perform the high- or low-demanding task with AR instructions. These tasks were randomly allocated to the participants in different sequences using a counterbalancing method to avoid the ordering effect of the tasks. The AB or BA sequences (A and B refer to high- and low-demanding tasks, respectively) were applied. In the AB sequence, half of the participants within group 1 were asked to perform a highly demanding task first (repairing a piston pump’s gearbox). The remaining participants were assigned to the BA sequence and were asked to perform the least-demanding task first. Two days later, participants returned to the lab to start the other half of the experiment, which is the task that was not performed in the first half (i.e., a low or high-demanding task), with AR instructions. Participants in group 2 (who used paper-based instructions) followed the same procedures as group 1.

Before the performance of each training task, the EEG electrodes were placed on the participants’ skin. After that, three minutes of EEG signals were recorded at resting postures to be used as baseline data. During the training tasks, EEG signals were recorded. After the training tasks were completed, the signals were recorded again for three minutes, and the training time was recorded. Total training time is the time taken to complete the task while following the instructions. The camera system and stopwatch were used to record this time. Upon completing the task, the electrodes were removed, and the participants were requested to complete questionnaires to measure their subjective perception of the task and the technique of completing it in terms of perceived workload.

### 2.8. Data Analysis

Statistical analyses were implemented using the SPSS Statistics software version 23.0 (IBM Corp, N.Y., USA). The significance level (type I error) was set to 0.05. The reliability of the statistical analysis was verified by checking the design assumptions of normality, homogeneity of variance, and continuity of data. To ensure that the normalcy assumption is true, a Kolmogorov-Smirnova test was performed [76]. A two-way analysis of variance (ANOVA) for the repeated measures design was implemented to test the main and interaction effects of the instruction method and task complexity on the dependent variables, including the power spectra density of EEG wave bands, perceived workload (NASA-TLX), and total training time. Significant interaction effects were further examined using simple effect analysis. The paired *t*-test was used for pairwise comparisons of the interaction effects of the task complexity levels. An independent sample *t*-test was performed for pairwise comparisons of the interaction effects of the levels of the instruction methods. The mean and standard deviation were calculated for all dependent variables. Furthermore, the effect size was determined by calculating the percentage of variance in the dependent variables that can be attributed to the specific independent variable using the partial eta-squared value (2).

## 3. Results

### 3.1. Performance

**Figure 6:**
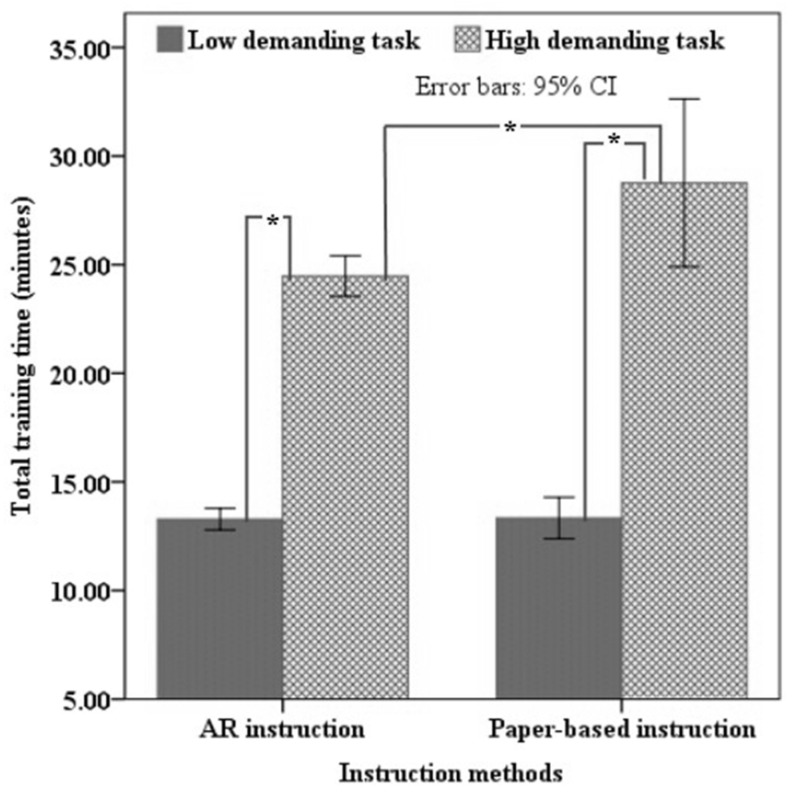
Average total training time for all levels of instruction methods and task complexity. Error bars represent a 95% confidence interval. Columns connected with lines and “*” marks are significantly different from each other.

The result of the mixed ANOVA revealed that there was a significant main effect of task complexity on the total training time (*p* < 0.05). The training time for maintenance tasks varied significantly, with a considerable rise from the low-demanding (mean = 13.37 min) to the high-demanding (mean = 26.62 min) tasks, regardless of the instruction methods. Furthermore, the training time was significantly affected by the interaction between instruction methods and task complexity (*p* < 0.05). Based on simple effect analysis (Figure 6), the results revealed that the training time during the high-demanding task was significantly higher than the low-demanding task in both the AR-based instruction method (24.48 vs. 13.30 min; *p* < 0.05) and the paper-based instruction method (28.76 vs. 13.36 min; *p* < 0.05). Additionally, the results revealed that for the highly demanding task, the completion time for the AR and paper-based instruction groups was significantly different (24.48 vs. 28.76 min; *p* < 0.05). The AR group also finished the task with low demand more quickly than the paper-based instruction group (13.30 vs. 13.36 min), but no statistically significant difference was found (*p* > 0.05). The most important result of this analysis is that performing a highly demanding task using AR instructions decreased training time.

### 3.2. Mental Workload

#### 3.2.1. Theta (θ) Power Spectrum Density

The statistical results shown in Table 3 revealed that the main effect of the instruction method was statistically significant on θ power for central and parietal regions (*p* < 0.05). The θ power at the central and parietal regions was found to be increased during the AR-based instruction task as compared to the paper-based instruction. However, the θ power at the frontal, occipital, and temporal regions was found to not be statistically affected by the instruction method (*p* > 0.05). The main effect of task complexity was statistically significant on θ power for the frontal, central, parietal, occipital, and temporal regions (*p* < 0.05). The θ power was found to increase as task complexity increased from a low to a high-demanding task for all regions of interest, regardless of the instruction methods used. For the interaction effects, the statistical results revealed that there was a significant interaction effect of instruction method and task complexity on θ power only at the parietal region (*p* < 0.03).

**Figure 7:**
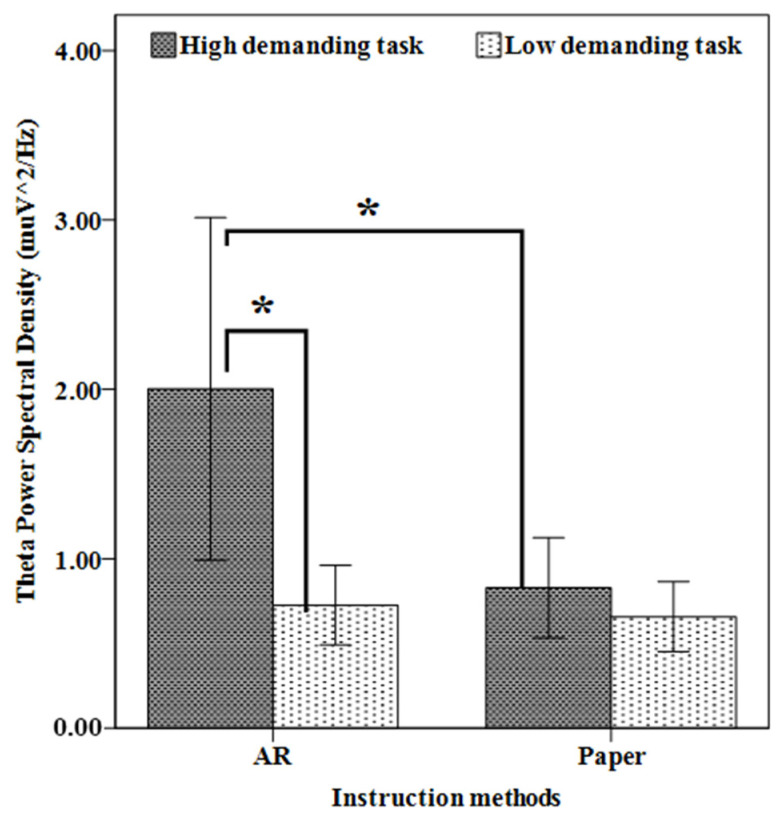
θ power for the parietal region for all levels of task complexity and instruction methods. Error bars represent a 95% confidence interval. Columns connected with lines and “*” marks are significantly different from each other.

Further analyses were performed using paired *t*-tests and independent *t*-tests to investigate the differences in θ power for the parietal region across maintenance task complexity and instruction methods (Figure 7). The results revealed that performing the high-demand maintenance task using AR instructions led to increased θ power as compared to the low-demand task (*p* < 0.05), but no significant difference was found between the high- and low-demand tasks when participants used paper-based instruction (*p* < 0.05). In addition, there was a significant difference between paper-based and AR-based instructions when participants performed the highly demanding task (*p* < 0.05). Furthermore, no significant difference was found between AR and paper-based instructions for the less demanding task (*p* > 0.05).

#### 3.2.2. Alpha (α) Power Spectrum Density

The instruction method was found to significantly affect α power at the frontal, central, parietal, occipital, and temporal regions (*p* < 0.05). The results revealed that wearing Microsoft HoloLens increased the α power at all regions of interest. Also, task complexity has significantly affected α power all regions of interest (*p* < 0.05). Specifically, the high complexity level resulted in higher α power values, regardless of the instruction methods. For the interaction effects, the statistical results revealed that there were significant interaction effects between instruction method and task complexity on α power of the frontal, central, parietal, occipital, and temporal regions (*p* < 0.05).

**Figure 8:**
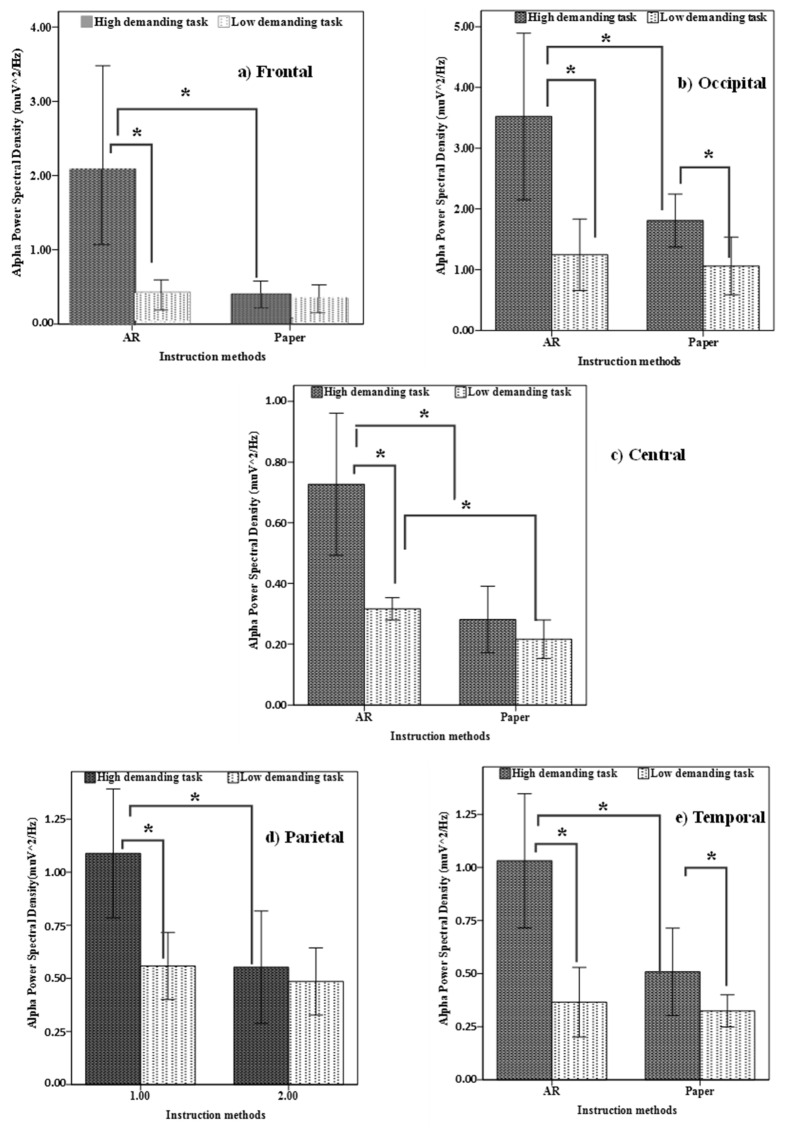
α power for (**a**) frontal, (**b**) occipital, (**c**) central, (**d**) parietal, and (**e**) temporal regions for all levels of task complexity and instruction methods. Error bars represent a 95% confidence interval. Columns connected with lines and “*” marks are significantly different from each other.

Further analyses were performed using paired *t*-tests and independent *t*-tests to investigate the differences in α power across task complexity and instruction methods for all regions of interest (Figure 8). For the AR method, the α power during the high-demanding task demonstrated significantly larger values than during the low-demanding task and during the high-demanding task of paper-based instruction across all regions of interest (*p* < 0.05). In addition, for paper-based instruction, the α power of the occipital and temporal regions for the high-demanding task increased significantly relative to the low-demanding task but remained approximately the same over the frontal, central, and parietal regions. Furthermore, the α power at the central region for AR groups was found to be significantly increased relative to paper-based instruction during the low-demanding task.

#### 3.2.3. Beta (β) Power Spectrum Density

The statistical results revealed that the main effect of the instruction method was statistically significant on β power for the central and occipital regions (*p* < 0.05). This effect resulted in significantly decreasing β power values during AR-based instruction as compared to paper-based instruction at the central and occipital regions. The remaining regions followed the same change pattern, but not significantly. The main effect of task complexity was statistically significant on β power for all regions of interest (*p* < 0.05). Less values of β power were observed as task complexity increased from a low to a high-demanding task for all regions of interest, no matter the instruction methods used. For the interaction effects, the statistical results revealed that there were no significant interaction effects between instruction method and task complexity on β power for all regions of interest.

#### 3.2.4. Perceived Workload (NASA-TLX) Scores

Perceived workload levels were assessed with the NASA-TLX workload questionnaire [37]. Increased scores on subscales indicate increased perceived demand, except for the performance subscale, which implies lesser self-reported task success. Table 4 summarizes mixed ANOVA results for NASA-TLX sub-scores. The effect of task complexity on the NASA-TLX overall score and the ratings of the six dimensions was statistically significant (*p* < 0.001). The ratings for all the dimensions were higher during the highly demanding task. The effect of an instruction method was found to be significant only on the ratings for performance and frustration (*p* < 0.001) and on the NASA-TLX overall score (*p* < 0.005), with these scores being higher for the paper-based instruction method (vs. AR-based). The interaction between task complexity and instruction method was found to be statistically significant on all the NASA-TLX subscales (*p* < 0.05) except effort and overall score (*p* > 0.05).

## 4. Discussion

The goal of this study was to find out how performance and cognitive workload are affected by different instruction methods (paper-based vs. AR-based instructions) and task complexity (high vs. low-demanding tasks) when using maintenance applications. Currently, monitoring the workload of AR system users is mainly subjective. Therefore, accurate and sensitive methodologies are required for assessing the workload of AR system users during maintenance operations. To the best of our knowledge, there are very few studies that integrate subjective and objective metrics to evaluate the mental workload of AR system users during maintenance operations. The work presented here focuses on how AR-based instruction methods with different levels of demanding tasks influence human performance and cognitive workload. The overall findings of this study mainly confirm the original hypotheses.

### 4.1. Performance

User performance while using an AR instruction method was assessed against the paper instruction method using total training time. Maintenance training times varied significantly between tasks, with a significant increase from low-demanding to high-demanding regardless of instruction method type (i.e., a 50% increase). The training time for the high-demanding task was significantly higher than the low-demanding task in the AR-based instruction method (24.48 vs. 13.30 min) and the paper-based instruction method (28.76 vs. 13.36 min). Using AR instructions, the time was saved by 0.45% for the low-demanding task and 14.94% for the high-demanding task as compared to paper instructions. The study’s findings revealed that using AR-based instruction during high-demanding tasks significantly speeds up maintenance training when compared to paper-based instruction. It could be argued that paper-based instruction for this highly demanding task is time-consuming as it requires browsing papers and extracting different features. In contrast, the introduced AR-based instruction provides different functionalities that can be presented on demand (i.e., virtual and real contents). On the other hand, no advantage in training time was observed while using AR for the low-difficulty task with a small number of task steps. Thus, AR instructions showed great improvement in training time as the difficulty level increased.

The findings of this study are in line with those in existing studies [31,77,78,79,80]. Wiedenmaier et al. [31] found that paper manuals were not significantly different from AR instruction when it came to simple activities, but when it came to more challenging jobs, the AR support proved to be better. Deshpande & Kim [81] discovered that utilizing AR in a simple assembly activity is less expensive than using paper instructions. Mengoni et al. [77] discovered that using AR for a simple job yielded similar results as using traditional techniques. Alves et al. [79] recommended that “augmented reality’s potential lies in its application to more complex tasks." Bendzioch et al. [80] evaluated the effect of different types of instructions (AR-based, tablet-based, projection-based, and paper instructions) and product complexity (difficult, medium, and easy) on total completion time. Their result showed that using AR glasses for instructions resulted in less completion time by 3.16% for easy, 9.51% for medium, and 9.00% for difficult products as compared to paper instructions [80]. 

### 4.2. Mental Workload

The mental workload can be perceived as the ratio of available cognitive resources to external and task-related demands. The cognitive processes that are connected with mental workload begin with data reception and processing and end with motor response activation and motor pattern monitoring [82]. The limits of human capacity, especially those of working memory, are a limiting factor [83,84]. Each decision-making process can be viewed as a unique cognitive operation characterized by the allocation of resources in a network of interconnected brain areas [85]. When there are more tasks to be performed, more resources must be utilized, and more of the available capacity must be put to use. Therefore, "mental workload" describes the amount of effort exerted by the brain in order to fulfill task demands [86]. 

### 4.3. Perceived Workload (NASA-TLX) Scores

Compared to the AR-based instruction, the average physical workload, performance, and frustration ratings were significantly higher when the participants performed the high-demanding task using paper-based instruction. Utilizing the AR instruction technique for highly demanding tasks led to a reduction of 12.18% in physical workload, 8.69% for performance, and 60.14% for frustration. In addition, when the participants performed the low-demanding task using AR instruction as compared to paper-based instruction, the average temporal demand ratings were significantly higher and performance ratings were lower. Compared to the paper-based method, using AR instruction for the low-demanding task led to lower performance and frustration ratings by 27.64% and 24.97%, respectively, and more temporal demand by 43.63%. Overall, the AR-based instruction task was found to cause a reduction of 18.13% for performance, 50.82% for frustration, and 6.50% for overall mean scores. Also, the average mental and physical workloads and temporal and frustration ratings for the two instruction methods were found to increase as the task complexity increased.

Previous research on the effect of AR tools on perceived workload and performance during training has yielded inconclusive results, with some researchers claiming that using AR to deliver visual guidance and task-relevant information reduced cognitive load and increased performance [32,87,88], while others claim that it is cognitively demanding and can lead to decreased performance [89,90]. The findings of this study are in line with those reported in [32,87,88]. Another study found that with AR assistance, mental activity associated with locating and interpreting needed information for a task is reduced, and only effort is required for task execution [91]. Also, Deshpande and Kim [81] found an increase in cognitive load but no change in task performance when AR was used. For example, Dan and Reiner [92] reported that processing 3D information is less taxing on the brain than processing 2D information. Relevance and timeliness of material, information presentation, user and task characteristics, and augmented reality display device type were demonstrated as factors that could have impacts on cognitive workload and task performance [92]. These different findings could be attributed to differences in the types of studies, the technology used, and the research designs that have been used.

### 4.4. EEG Power Spectrum Density

The results revealed that performing the high-demand maintenance task using AR instruction led to an increase in the theta power in the parietal region as compared to the low-demand task, but no change was detected between the high and low-demand tasks when participants used paper-based instruction. In addition, there was a significant change between paper-based and AR instructions while participants performed the most demanding task, but no significant difference was found between the least demanding tasks. The increased theta power at the parietal region, when the participants performed the highly demanding task using AR instruction, may be an indicator of attentional orienting to a task-relevant class of event. On the other hand, participants who performed the high- and low-demanding tasks using paper-based instruction used the same class of cues for processing the information. Related to α power, the findings revealed that for the AR method, the α power during the high-demanding task demonstrated significantly larger values than during the low-demanding task across all regions of interest. In addition, for paper-based instruction, the α power of the temporal and occipital regions for the high-demanding task increased significantly relative to the low-demanding task but remained approximately the same over the frontal, central, and parietal regions. The results showed that at a low-demanding task, the α power in the central region for AR groups was found to be significantly increased relative to paper-based instruction but not significantly increased in the parietal, frontal, temporal, and occipital regions. These increases in alpha power are possibly due to sensory inhibition or internal processing demands. The drastic increase in alpha power attributed to the execution of the mental demand during the AR experience is remarkable. When compared to paper-based instruction, β power was found to be reduced in the central and occipital regions during AR instruction. In addition, the β power values were found to decrease as task complexity increased for all regions of interest, no matter what the instruction methods used. The increase of theta and alpha and the decrease of beta have probably resulted from the perceived intrinsic load that participants interpret as the intricacy of the objective affordances concerning their abilities to handle the maintenance task (i.e., their prior knowledge). As the task load increases, so does the amount of resources used, and more of the available capacity is used [86].

The findings of this study are in line with previous studies, which reported that θ and α activities may be associated with memory, cognition, and attention [54,93]. θ was also linked with other mental processes and workload [64]. The AR instruction for the highly demanding task used more visual cues to orient focus toward the necessary steps in the AR environment, which led to enhanced target stimulus processing speed and accuracy. Increased frontal activations for AR relative to paper instruction could be understood as associated with inwardly driven decision-making processes about where to attend [94,95,96]. Moreover, during creativity tasks, the alpha power of the frontal, parietal, occipital, and right hemispheric regions was reported to be increased [97,98,99,100,101]. The changes in alpha seem to be more inwardly focused, which could be attributed to attention [102]. The theta band also tends to increase when one is focused inward and experiencing pleasant emotions [103]. Similar to how alpha activity plays a different function in internal vs. external attention, theta activity does the same [104]. Therefore, our findings regarding the synchronization of alpha and theta waves may be regarded as increased cortical activation during internal focus of attention. Cooper et al. [105] discovered that alpha power increased with task demands and was highest when participants focused their attention internally on mental imagination tasks rather than externally on sensory intake activities. Higher alpha values may indicate cognitive process inhibition that is not directly related to task performance and is observed through positions that are most likely to be subject to or to impose top-down control [106]. 

The overall findings of this study revealed that the use of AR instruction caused an increase in mental workload. Such an increase could be interpreted as a more germane cognitive load, which is desirable as it is associated with learning new skills and other information. So, AR fosters the construction of schemata (GCL) and mental models by activating the cognitive processes of the participants and encouraging them to invest more effort in learning. The germane load during AR is increased due to incorporating new information, as well as the development and updating of schema, which takes up some of the memory [18]. To aid in learning and schema development, augmented reality (AR) adopted the concept of cognitive activation to focus the user’s attention on the most pertinent cognitive processes [18]. The findings indicated that the AR system could enhance the cognitive element of learning; by connecting virtual objects to the real-world setting, AR enables actual and virtual objects to display themselves in a single spatially embedded view, which is in line with the previous study by [107]. The structure of the information presented in AR is compatible with the users’ inherent mental models [108]. In addition, AR consequently helps to integrate information: each relationship between a virtual object and a real-world object is encrypted and stored as a single visual picture forming the foundation for a memory connection [109]. Future research should assess this interpretation by comparing how much knowledge is transferred when using paper-based instructions to do a complex task versus AR-based instructions.

Furthermore, the increased mental workload during AR usage may arise from the fact that delivering AR instructions is a novel method and participants have to learn new interaction techniques, which activates the cognitive processes of the participants to invest more effort into learning, raising mental effort. Hence, using AR as an instruction method, participants could better facilitate long-term knowledge and skill acquisition, manage their working memory load, and learn successfully. During the AR instruction method, the information is processed in working memory and stored in long-term memory, and knowledge, in accordance with schema theory, is stored and arranged in the mind in the shape of schemata. Also, during AR, the participants spent a greater amount of their mental resources on processes that were irrelevant to the actual task, like repressing instruction through animation, visual cues, and arrows. To overcome this, it is preferable to show an instruction visually when put together with animation. While presented instruction combined with animation requires visual resources, auditory instruction uses the phonological system of working memory [83]. Another possible explanation for the increased mental workload during AR may result from task difficulties [110]. Moreover, the increased cognitive workload when performing AR-guided activities may be due to focus rivalry (FR), which happens when simultaneously concentrating real and digital information positioned in the user’s immediate personal surroundings [111], restricting their usage in high-precision manual tasks [11,110,112].In order to mitigate the increased mental workload observed with AR instruction, several strategies could be employed. Firstly, the AR system could be adaptively designed to match the user’s pace and learning style, thus customizing the learning experience. Secondly, a multimodal instruction approach utilizing both auditory and visual cues could be implemented, which could distribute the cognitive load across different channels. Thirdly, providing preliminary training sessions could help familiarize users with the AR system and its interaction techniques before they undertake complex tasks. Fourthly, an emphasis on user-friendly interface design, including clear icons, intuitive navigation, and easy-to-understand instructions, could minimize unnecessary cognitive load. Lastly, scheduling regular breaks during AR instruction could aid in preventing cognitive overload. These strategies collectively aim to enhance the efficacy of AR as an instructional tool by effectively managing cognitive load and warrant further research for refinement and efficacy testing.

The current study has some limitations that are worth noting. The design was limited to a standing posture. The participants were restricted to standing during the task performance. Therefore, the effects of different postures (e.g., sitting and standing) should be investigated in the future. Second, upper body kinematics were not monitored in this study. Tracking inter-individual kinematic differences may aid in explaining observed differences in stiffness responses to complex tasks and instruction methods. In addition, all participants were male students. Future works will include female and other physiological signals (i.e., autonomic nervous system and upper arm muscle activity). Moreover, the target users were also novices, so it would be better to recruit experienced participants or experts to make the evaluations and comparisons more complete. Furthermore, the range of the participants’ ages was quite narrow, so it would be interesting to do more research on a broader age group in the future. The results and conclusions of this study were reached in light of the particular interfaces utilized for this user study (visual cues with animation), and the usage of different variations of the interface (visual with auditory), technology (HMD, HHD), and research designs (between, within, or mixed design) may yield different results and conclusions. Finally, this study also illustrated very effectively that the benefits promised by AR for maintenance applications will not be realized automatically.

## 5. Conclusions

This study evaluated the effects of instruction methods and task complexity on cognitive and perceived workloads in maintenance applications. The results supported the hypotheses that the AR method is more suitable, efficient, and effective to display maintenance instructions than the paper method; that AR instructions lead to faster maintenance times than paper instructions; and that AR instructions lead to a greater increase in mental workload than paper-based instructions, especially for the highly demanding task. Using AR instructions, the time was saved by 0.45% for the low-demanding task and by 14.94% for the high-demanding task as compared to paper instructions. It is argued that paper-based maintenance instruction for the highly complex task is time-consuming (i.e., viewing a paper and extracting different features). In contrast, the introduced AR-based instruction provides different functionalities that are presented on demand. Thus, the benefit of using AR for the low-difficulty task (with a small number of task steps) was relatively small. As the difficulty level increased, the advantages of applying AR to an instruction system became more obvious. Thus, in similar cases of higher task complexity, AR is recommended to be utilized. Furthermore, AR could enhance cognitive abilities to process information by easing information access and keeping working memory capacity free for use. Overall, the use of AR to guide workers through high-demanding maintenance tasks increased information processing, which could be connected to germane cognitive load due to learning. The findings suggested that AR instruction increased the germane cognitive load, which is the portion of cognitive load that assists in learning new skills and other information. AR for a high-demanding task allows for using cognitive or metacognitive prompts to hustle the participant in the right direction or to give the participant cues on how to best process the maintenance instructions. So, AR fosters the construction of schemata (GCL) and mental models by activating the cognitive processes of the participants to invest effort into learning, raising mental effort.

Overall, this study shows that augmented reality should be used for high-demand maintenance tasks because it cuts down on maintenance time and makes it more likely that information will be stored in long-term memory and encrypted for later use. The results of this study can help manufacturing stakeholders better understand the benefits of AR technology as an instruction tool for manual tasks. Furthermore, the findings of this study could aid manufacturers of AR instruments in fine-tuning their products to overcome some of the limitations encountered. Also, the results of this study are important for the advancement of knowledge relative to cognitive workload evaluation while using AR systems in maintenance and assembly tasks.

## Figures and Tables

**Table 1:** List of the maintenance steps for the high-demanding task (piston pump).

	Disassembly Steps		Assembly Steps
1	Detach the V-belt pulley from the crankshaft.	21	Before installing the grooved ball bearings on the crankshaft, lightly lubricate the shaft.
2	Remove the stud bolt, hexagon nut, and washer.	22	Fit the ball bearing with the groove at the connecting rod seat on the crankshaft.
3	Pull the packing gland out of the pump and remove the hexagon nut from the stud bolts.	23	Without bending the crankshaft, drive the grooved ball bearing onto it using a soft hammer, until the grooved ball bearing hits the recess.
4	Detach the pump housing from the gearbox. Carefully guide the piston out of the cylinder pipe when doing so.	24	Insert the piston rod and connecting rod into the gearbox. The crosshead should not be inserted into the guide bush just yet; the piston rod should protrude slightly from the oil splash guard.
5	Remove duo’s hexagon nut with the spring ring from duo-piston	25	Install an oil splash guard in the housing of the gearbox.
6	Pull the duo-piston from the piston rod.	26	Twist the oil stripper and press it against the piston rod.
7	Remove the hexagon nut from the rod.	27	Position the packing gland seal on the packing gland.
8	Remove the open bearing cover and bearing cover seal from the gearbox housing by unscrewing the hexagon screws.	28	Slot the piston rod through the packing gland and insert the crosshead in the guide bush.
9	Remove the closed bearing cover and bearing cover seal from the gearbox housing by unscrewing the hexagon screws.	29	Screw a hexagon nut and washer onto the piston rod.
10	Lever gearbox cover from the gearbox housing	30	Insert the duo-piston onto the piston rod.
11	Loosen screw fitting on connecting rod	31	Tighten the hexagon nut with the spring.
12	Pull the crankshaft from the base of the connecting rods. To remove the connecting rod bearing, you must first spread the base of the rod using a screwdriver.	32	Fit crankshaft into connecting rod base.
13	Take the crosshead and the rod that controls the piston out of the guide bush. The connecting rod should be left inside the gearbox.	33	To facilitate the insertion of the connecting rod bearing, open up the base of the rod using a screwdriver.
14	Take packing gland seal from packing gland.	34	Mount the gearbox cover on the gearbox housing.
15	Twist the oil stripper off the piston rod.	35	Position the bearing cover seal against the gearbox housing.
16	Lever oil splash guard from the gearbox housing	36	Drive hexagonal screws into the gearbox housing to secure the open bearing cover.
17	Take the connecting rod and piston rod out of the gearbox housing.	37	Press the gearbox housing against the pump housing.
18	Insert the puller into the ball bearing groove. The hook arms are evenly pressed against the ball-bearing grooves.	38	Connect the pump’s gearbox and housing using the hexagon nut.
19	Turn the adjuster thread against the shaft end. Ensure the jig is centered.	39	Put packing glands on stud bolts using a hexagon nut and washer.
20	Carefully turn the adjustment thread to remove the grooved ball bearing from the crankshaft.	40	Push the V-belt pulley onto the crankshaft.

**Table 2:** List of the maintenance steps for the low-demanding task (pump housing).

	Disassembly Steps		Assembly Steps
1	Unscrew the water plug with the seal from the air chamber.	14	Place the valve seat seal into the pump housing.
2	Unscrew the cap nut and washer from the air chamber bolt.	15	Place both intake valves on one side of a pump housing.
3	Lift the air chamber from the pump housing.	16	Position both pressure valves on the pump housing’s opposite side.
4	Remove the air chamber seal from the housing.	17	Cover each pressure and intake valve with a bridge.
5	Remove the bolt for the air chamber from the pump housing.	18	Use a hexagonal screw and washer to secure the valve bridges.
6	Loosen hexagon screws and take off the intake and pressure flanges with seals.	19	Tighten the drain plug containing the seal into the tapped hole.
7	Take off the drain plug and seal it from the tapped hole in the pump housing.	20	Fit seals at intake and pressure fittings.
8	Remove the hex nuts and washers from the valve bridge stud bolts.	21	Tighten both straight flanges with hexagon screws to the intake and pressure fittings.
9	Raise valve bridges from intake and pressure valves.	22	Screw the air chamber bolt into the pump housing.
10	Take the pressure valve out of the pump housing.	23	Place the air chamber seal on the housing by pushing it over the air chamber bolt.
11	Take the intake valve out of the pump housing.	24	Attach the air chamber to the pump case. The intake valves must be located above the membrane safety valve opening.
12	Remove the valve seat seal (59) from the pump housing.	25	Screw a cap nut with a washer onto the air chamber bolt.
13	Check valve seat seals and packing gland seals.	26	Screw the water plug with the seal into the air chamber.

**Table 3:** Mean (SD) and statistical results for θ, α, and β power of all regions of interest (i.e., Frontal, central, parietal, occipital, and temporal) across experimental tasks. Bolded *p*-value indicates a significant effect. Note: η2 is partial eta squared.

	Variable	Mean (SD)				*p*-Value (η2)		
	Instruction Methods	Paper-Based Instruction		AR-Based Instruction		Instruction	Complexity	Interaction
Brain Regions	Task Complexity	High	Low	High	Low			
Frontal	Theta (θ)	0.99 (0.98)	0.69 (0.61)	1.26 (0.77)	0.74 (0.41)	0.54 (0.02)	**0.04 (0.21)**	0.59 (0.02)
	Alpha (α)	0.41 (0.23)	0.35 (0.25)	2.08 (1.95)	0.43 (0.22)	**0.01 (0.30)**	**0.01 (0.30)**	**0.02 (0.30)**
	Beta (β)	0.21 (0.04)	0.27 (0.11)	0.15 (0.07)	0.25 (0.06)	0.12 (0.13)	**0.002 (0.43)**	0.51 (0.024)
Central	Theta (θ)	0.53 (0.29)	0.47 (0.19)	1.09 (0.66)	0.47 (0.19)	**0.01 (0.32)**	**0.003 (0.4)**	0.08 (0.16)
	Alpha (α)	0.28 (0.15)	0.22 (0.09)	0.73 (0.33)	0.31 (0.05)	**0.00 (0.50)**	**0.00 (0.54)**	**0.00 (0.40)**
	Beta (β)	0.20 (0.06)	0.35 (0.16)	0.11 (00.03)	0.16 (0.8)	**0.00 (0.52)**	**0.00 (0.41)**	0.12 (0.13)
Parietal	Theta (θ)	0.83 (0.41)	0.66 (0.28)	2.00 (1.41)	0.73 (0.33)	**0.02 (0.26)**	**0.00 (0.34)**	**0.03 (0.23)**
	Alpha (α)	0.55 (0.37)	0.49 (0.22)	1.09 (0.43)	0.56 (0.22)	**0.01 (0.30)**	**0.00 (0.40)**	**0.02 (0.30)**
	Beta (β)	0.57 (0.32)	0.61 (0.19)	0.34 (0.15)	0.60 (0.21)	0.20 (0.11)	**0.03 (0.24)**	0.10 (0.15)
Occipital	Theta (θ)	2.45 (1.43)	1.5 (1.05)	2.79 (1.55)	1.21 (0.84)	0.96 (0)	**0.00 (0.51)**	0.3 (0.06)
	Alpha (α)	1.81 (0.61)	1.060 (0.67)	3.52 (1.91)	1.24 (0.82)	**0.03 (0.25)**	**0.00 (0.54)**	**0.03 (0.23)**
	Beta (β)	2.20 (0.63)	2.67 (0.55)	1.42 (0.38)	1.60 (0.37)	**0.00 (0.55)**	**0.00 (0.35)**	0.20 (0.10)
Temporal	Theta (θ)	0.66 (0.45)	0.50 (0.24)	0.92 (0.34)	0.44 (0.23)	0.4 (0.043)	**0.00 (0.40)**	0.11 (0.14)
	Alpha (α)	0.51 (0.29)	0.32 (0.11)	1.03 (0.44)	0.36 (0.23)	**0.02 (0.26)**	**0.00 (0.70)**	**0.00 (0.43)**
	Beta (β)	0.27 (0.17)	0.36 (0.18)	0.18 (0.10)	0.34 (0.12)	0.35 (0.05)	**0.00 (0.40)**	0.35 (0.05)

**Table 4:** Mean (SD) and statistic results for perceived workload (NASA-Tlx) scores across experimental tasks. Bolded *p*-value indicates a significant effect. Note: η2 is partial eta squared.

Variable	Mean (SD)				Statistics *p* (η2)		
Instruction Methods	AR-Based Instruction		Paper-Based Instruction		Instruction (η2)	Complexity (η2)	Interaction
Task Complexity	High	Low	High	Low			
Mental demand	68.21 (13.10)	30.71 (11.24)	73.93 (7.12)	25.00 (11.24)	1 (0.00)	**0.00 (0.97)**	**0.00 (0.39)**
Physical demand	61.79 (7.50)	33.57 (8.64)	70.36 (7.71)	30.35 (8.43)	0.17 (0.07)	**0.00 (0.90)**	**0.02 (0.20)**
Temporal demand	66.79 (13.95)	40.00 (9.61)	66.79 (6.96)	27.85 (9.55)	0.07 (0.13)	**0.00 (0.90)**	**0.01 (0.21)**
Performance	75.00 (7.33)	58.92 (14.17)	82.14 (7.26)	81.43 (7.95)	**0.00 (0.50)**	**0.00 (0.40)**	**0.00 (0.33)**
Effort	69.29 (7.56)	48.93 (11.12)	68.93 (9.03)	58.21 (8.823)	0.07 (0.12)	**0.00 (0.60)**	0.06 (0.13)
Frustration	18.93 (6.56)	12.86 (5.08)	47.50 (9.35)	17.14 (5.08)	**0.00 (0.73)**	**0.00 (0.83)**	**0.00 (0.68)**
Overall weighted demand	68.35 (4.55)	43.33 (4.01)	71.48 (4.02)	47.95 (4.06)	**0.00 (0.26)**	**0.00 (0.96)**	0.43 (0.02)

## Data Availability

All data supporting reported results are included in the manuscript.
